# Supplementary material for: Deletion of wfs1 Impairs Oligodendrocyte Precursor Cells Dorsal Distribution and Myelination Through the wfs1-hmgcs1 Axis in Zebrafish
Source: Biology (Basel). 2026 Aug 16;15(16):1402. doi: 10.3390/biology15161402 (PMC13510151; doi:10.3390/biology15161402)
Supplement: Supplementary file 1 [file biology-15-01402-s001.zip › File S1-Transcriptome sequencing data and differentially expressed genes (DEGs) in wfs1-- zebrafish at 5 dpf/2.KEGG/wfs1KOvsWT/ALL/src/dre00534.html]

dre00534


- dre:791107

- Up regulated genes

791107\_1(1.62699683108684)

- dre:791107

- Up regulated genes

791107\_1(1.62699683108684)

- dre:792524

- Up regulated genes

792524(2.23418633181462)

- dre:792524

- Up regulated genes

792524(2.23418633181462)

- dre:792524

- Up regulated genes

792524(2.23418633181462)

Close
